# Genomic relationships, diversity and admixture in the Lucerna breed of Colombia

**DOI:** 10.1007/s11250-026-05227-y

**Published:** 2026-08-10

**Authors:** Maira Alejandra Montoya-Bedoya, Juan Carlos Rincón

**Affiliations:** Palmira Zoogenetic Resources Research Group, Department of Animal Science, Universidad Nacional de Colombia, Palmira, Valle del Cauca, Colombia

**Keywords:** Bovine ancestry, Creole bovines, Genetic resources, Genetic variability

## Abstract

The Valle del Cauca region of Colombia is the homeland of the Hartón del Valle (HV) breed, which has been adapted to lowland tropical conditions for more than 500 years. The Lucerna breed is a Colombian synthetic breed composed of 40% Holstein, 30% Shorthorn and 30% HV. Therefore, this study examined the genetic diversity, admixture, and relationships of the Lucerna breed. Information was collected from the farms where the breed originated: El Hatico Nature Reserve and Hacienda Lucerna. Additionally, the HV conservation nucleus (CN) at the National University of Colombia in Palmira was included for comparison and to assess the status of the CN. Genealogical information was collected from 9,526 Lucerna and 64 HV individuals. DNA was extracted from 94 Lucerna and 10 HV animals. The GGP-Bovine 100 K chip was used, quality control was applied (Excluding SNPs with a MAF < 0.05 and missing data > 0.05), to estimate minor allele frequencies (MAF) and LD. ROH fragments, Ne, heterozygosity and Fis were estimated, PCA analysis, population structuring by Fst. Admixture (K = 2 to 12) and Treemix between breeds and with additional information from other breeds. The average MAF was 0.31 for Lucerna and 0.28 in HV-CN. The observed and expected heterozygosity was higher in Lucerna. The average ROH coverage was 8.43% for HV-CN and 7.00% for Lucerna, with a higher proportion of recent autozygosity in both breeds. Finally, genetic diversity was lower in HV-CN than in Lucerna, which coincides with FAO reports. Analyses show that Lucerna has its own genomic profile.

**Supplementary Information:**

The online version contains supplementary material available at https://doi.org/10.1007/s11250-026-05227-y.

## Introduction

Creole cattle breeds are of significant genetic importance due to their role as a reservoir of genetic diversity, enabling adaptation to diverse environmental conditions. This ability enables them to resist diseases and develop specific abilities to make the best use of available resources. They are therefore a valuable resource for addressing the future challenges of climate change (Martínez Correal 2010). The characterization of these breeds allow us to appreciate their attributes in lowland tropical production systems, which encourages the development of conservation and multiplication programs (Bruford et al. 2015).

There are 10 breeds of Creole cattle in Colombia which have been adapted to different regions of the country. The Harton del Valle (HV) breed originates from cattle introduced to Valle del Cauca from the south of the country during the colonial conquest. Crossbreeding and selection processes were then employed to develop unique characteristics to enable the breed to adapt to tropical conditions and enhance its productivity in the agroecosystems of Valle del Cauca. The Lucerna breed was later created in Valle del Cauca. This synthetic breed is the result of crossbreeding the Holstein (40%), Shorthorn (30%) and HV (30%) cattle, and was developed as an alternative for milk production in the dry tropics (Martínez et al. 2019). The Lucerna breed has been established on the territory for more than 86 years. During the formation of the breed through inbreeding, it is unclear what proportion of its genes have been fixed in the population over the generations, and whether genetic findings can be used to determine whether it can be considered a breed. However, it has already been recognized as a breed by the FAO (FAO - Food and Agriculture Organization of the United Nations 2019). Despite the qualities of these two breeds that contribute to the country’s food security, little is known about their diversity, genetic history, population structure and many more components for their efficient breeding and use.

In recent decades, livestock breeding has advanced significantly in the field of genetics. The constant quest to improve cattle breeds’ productivity, efficiency and adaptability has led to the development of new tools and technologies that enable more in-depth analysis of their genetic composition. These advances have great potential for genetic improvement and the conservation of the adaptive traits of livestock breeds, such as Creole cattle (Ajmone-Marsan et al. 2023). Genomics has been presented as a potential tool for the improvement of Creole cattle. The utilization of genetic information contained within the genome of these animals enables the accurate estimation of diversity, admixture, and ancestry. This tool enables farmers to understand Creole cattle populations, identify desirable traits and conserve the unique adaptive traits of these breeds. Genomics also facilitates informed decision-making in breeding programs, which contributes to the selection of animals with higher genetic potential, thereby increasing the efficiency and sustainability of livestock production (Ajmone-Marsan et al. 2023). The objective of this study was to investigate genetic diversity, population structure, ancestry and genetic relationships in Lucerna and HV cattle in Colombia.

## Materials and methods

### Animals and DNA extraction

A total of 104 animals were considered for this study, of which 94 individuals were Lucerna (20 males and 74 females) from the farms Reserva Natural El Hatico and Hacienda Lucerna, and 10 Hartón del Valle animals (4 males, 6 females) from the conservation nucleus (HV-CN) at the Mario González Aranda farm of the National University of Colombia, Palmira, which were used for comparison and to assess the diversity present in the nucleus. Given the sample size, it is important to note that the aim is not to draw conclusions about the entire HV population. The animals were selected considering as criteria the contrasting genetic values for PTL and the average genealogical relationship of the individuals, to include animals with extreme genotypes and from the base of the population, in order to reflect the genetic diversity. Blood samples were collected for animals that were present, and semen for in-use bulls that were no further in the herd. Blood was collected using 21-gauge needles and BD Vacutainer tubes containing EDTA as an anticoagulant, by puncturing the coccygeal vein. DNA extraction was performed in the molecular biology laboratory at the National University of Colombia in Palmira using the “HigherPurity™ Blood DNA Extraction Kit” (Canvax^®^, Córdoba, Spain), following the manufacturer’s instructions. The samples were suspended in 100 µl of elution buffer. Using a COLIBRI spectrophotometer (Titertek Berthold^®^, Germany), the concentration and absorbance ratios A260/A280 and A260/A230 were analyzed to ensure values close to 1.8 and 2.0, respectively. Integrity was assessed by 1% agarose gel electrophoresis and the DNA was stored at -20 °C. Finally, DNA samples were lyophilized and sent for genotyping to the Neogen laboratory in Lansing, Michigan, USA.

### Genotyping

A total of 94 Lucerna (LUC) and 10 HV animals were genotyped. Genotyping was performed using the GGP-Bovine 100 K chip (90349 SNPs per animal). The genotypes obtained were subjected to a series of editing procedures. This included the removal of INDELS and SNPs in the mitochondrial and sex chromosomes using the R software (R Core team 2025). The Lucerna and HV-CN databases were then split for analysis. A series of quality control processes were applied using the PLINK v.1.90 program (Purcell et al. 2007) in accordance with the FAO’s suggested quality control process for genomic data (Ajmone-Marsan et al. 2023). First, genotypes and then individuals with missing data greater than 0.05 were excluded (--geno and --mind commands). Markers with a minor allele frequency (MAF) lower than 0.05 were also removed (--maf). Since we expected Hardy–Weinberg deviations in the studied populations due to their small and possibly sub-structured population, Hardy–Weinberg equilibrium SNP filtering was not performed.

### Linkage disequilibrium (LD), diversity and effective population size (Ne)

In the initial phase, a calculation of the minor allele frequency (MAF) for each genetic marker for each breed was undertaken. This was done after the application of the above quality control edition, using the PLINK v1.90 software (specifically the --freq command). R software (R Core team 2025) was used to evaluate the average and distribution of MAF values. LD analysis was performed with the r^2^ statistic between pairs of SNPs. This analysis was conducted using PLINK v1.90 software (ld-window-r2 0) (Purcell et al. 2007), was evaluated at a distance of 1,000 kb and plotted against distance in base pairs to observe LD decay using the ggplot2 package (Wickham 2017) in R software (R Core team 2025). The average r^2^ and the average distance at which an r^2^ greater than 0.3 is reached were estimated. Subsequently, SNeP v1.1 software (Barbato et al. 2015) was used with the PLINK files to estimate the effective population size (Ne), following the equation proposed by Corbin et al. (2012), a conversion of 1,000 kb = 1 cM was assumed. The analysis was based on the relationship between LD distribution and recombination rate.

### Runs of homozygosity (ROH) and inbreeding analysis

The ‘--homozyg’ and ‘--homozyg-group’ options of PLINK v1.90 software were used to identify Runs of Homozygosity (ROH) in the genome, for each breed, using a sliding window approach. In accordance with the approach outlined by Ferenčaković et al. (2013), the definitions of runs of homozygosity (ROH) incorporated the following parameters and thresholds: a sliding window of 50 SNPs across the genome (--homozyg-windows-snp), a homozygous window overlap ratio of 0.05 (--homozyg-windows-threshold), a minimum segment length of 1,000 kb (--homozyg-kb), a maximum gap of 1,000 kb between consecutive homozygous SNPs (--homozyg-gap), a minimum density of one SNP per 100 kb (--homozyg-density), a minimum of 50 SNPs per segment (--homozyg-snp), the maximum number of missing genotypes permitted was two (--homozyg-window-missing) and no heterozygous genotypes were allowed (--homozyg-window-het). The ROH segments identified were categorized according to length as follows: 1,000–4,000 kb, 4,000–8,000 kb, 8,000–12,000 kb, and > 12,000 kb. The inbreeding coefficient for ROH (FROH), as proposed by Bjelland et al. (2013), was then estimated using a genome length of 2,489,385.779 kb, which corresponds to the specified reference genome (ncbi.nlm.nih.gov/assembly/GCA_002263795.2). The length of the autozygous segments was assumed to follow an exponential distribution with mean t = ½g Morgans, where g is the number of generations since a common ancestor (Howrigan et al. 2011), which, in the case of segments larger than 12,000 kb, would indicate the last 4.17 generations.

### Diversity analysis

The Lucerna and HV-CN databases were then pruned using the ‘--indep-pairwise’ command in PLINK v1.90 software (Purcell et al. 2007), with parameters set to 50, 5 and 0.2. This indicates windows of 50 SNPs, with a sliding window of 5 SNPs and an r² threshold of 0.20, in order to maintain informative markers and reduce the overrepresentation of certain regions of the genome. Once this had been completed, the observed and expected heterozygosity for both breeds were calculated using the ‘--het’ command in PLINK v1.90 (Purcell et al. 2007), according to the formula proposed by Keller et al. (2011). Additionally, the inbreeding coefficient was estimated using the fixation index (FI) based on heterozygosity (HET), by comparing expected (He) and observed (Ho) heterozygosity.

### Multibreed analysis

In addition to the Lucerna and HV-CN genotyping mentioned previously, information was included from 25 Brahman, 24 Gir (Matukumalli et al. 2009), 30 Holstein (Gautier et al. 2010), 32 Negra Andaluza (Flori et al. 2019), eight Romosinuano and 34 Shorthorn (Decker et al. 2014) individuals. These genetic data were obtained from the WIDDE database (http://widde.toulouse.inra.fr/widde). The data for Brahman, Gir and Holstein were obtained from the Illumina BovineSNP50 v1 chip, and the data for Negra Andaluza (NAN), Romosinuano and Shorthorn were obtained from the Illumina BovineSNP50 v2 chip. This additional genetic information was used to identify genetic structure, diversity, and ancestral admixture relationships. The analysis included the HV, Holstein and Shorthorn breeds to determine their percentage composition within the Lucerna breed, the Brahman and Gir breeds to identify potential gene mixtures given their recent widespread use in the country, the Negra Andaluza breed due to its ancestral link to cattle introduced during the conquest era, and the Romosinuano breed, another Colombian Creole breed which shares a common ancestry and served as a control for database merging. The analysis was carried out in two stages: (i) analyzing the breeds that directly compose the Lucerna breed (i.e. the Hartón del Valle, Holstein and Shorthorn breeds); (ii) extending the analysis to include the other breeds to obtain a more complete picture of the Lucerna breed’s genetic composition and ancestral admixture relationships with other cattle breeds in a principal component analysis (PCA).

The databases were merged using the v1.90 PLINK software to identify common genetic variants, with forward allele coding being used. Since both genotyping platforms are Illumina, the databases contained 30,638 SNPs in common. However, despite their similarity and use of the same map, some variants with three or more alleles were found during the merging process (--bmerge). This indicates a problem with the DNA strand used as a reference. In these cases, the ‘--flip’ function was used to swap the alleles, considering the ancestral allele reported in the genomic map. To ensure proper swapping, allele frequencies were estimated by breed for comparison. Additionally, the Romosinuano breed was used as a merge control. The process was subjected to meticulous scrutiny using the R program, resulting in the removal of single-nucleotide polymorphisms (SNPs) where the appropriate merging procedure was ambiguous. Following this, 79,885 genetic variants were identified for Lucerna and HV-CN, with a genotyping rate of 99.4%; 26,305 common variants were found for the merge with the Holstein and Shorthorn databases, with a genotyping rate of 99.68%; and 25,074 SNPs for all breeds, with a genotyping rate of 99.59%. Database merging represents a significant portion of the original data. This is especially true when considering that LD pruning must subsequently be performed for population analyses and that pruning results in a significant reduction in genotyping density.

To maintain informative markers and reduce the overrepresentation of certain regions of the genome, the merged database was LD-pruned using the --indep-pairwise function of PLINK v1.90 (Purcell et al. 2007), with parameters set to 50, 5, and 0.2. These parameters indicate windows of 50 SNPs, with a sliding window of five SNPs and an r² threshold of 0.20. After quality control, the database of breeds that directly composed Lucerna included 10,877 SNPs and 101 individuals, while the database including all breeds consisted of 12,404 SNPs and 190 individuals. For some analyses, such as principal component analysis (PCA), a random subsample of the Lucerna breed was used to reduce bias due to its overrepresentation. Then, a principal component analysis (PCA) was performed on both databases using the --pca command in PLINK v1.90 (Purcell et al. 2007), and the results were visualized with the R package ggplot2 (Wickham 2017; R Core team 2025).

### Genetic structuring, admixture and treemix analysis

We used the all-breed LD-pruned database to visualize the genetic structure of the studied populations and examine the relationships between individuals. Minor allele frequencies (MAF) were estimated for each breed using the ‘--freq’ command with the ‘—family’ option in PLINK v1.90. Two genetic distances were included in the analysis. First, a genomic distance matrix (IBS) was generated using the ‘snpStats’ package (Clayton 2025) in R version 4.3 (R Core team 2025). Secondly, Nei genetic distances were calculated using the ‘poppr’ package (Kamvar et al. 2015) in R version 4.3. The phylogenetic tree was then constructed using the Nei distance matrix with the ‘factoextra’ package (Kassambara and Mundt 2020) to illustrate the genetic relationships between the breeds. The IBS distance matrix was represented using a dendrogram and a heatmap with the ‘heatmap’ function. The Arlequin files were obtained from the Plink files using the PGDSpider 2.1 program (Lischer and Excoffier 2012). The populations were defined according to breed (Brahman, Gir, Holstein, Harton del Valle, Lucerna, Negra andaluza, Romosinuano and Shorthorn), with all breeds represented. The Slatkin FSTs were calculated using the Arlequin software (Arlecore for Linux) (Excoffier and Lischer 2010). The number of migrants per generation (Nm) needed to maintain that population structure, was estimated based on the following expression derived from Wright’s statistics, Nm=[(1/FST)-1]/4.

In order to detect population structure based on Nei genetic distances, the “NetvieweR” package (Steinig et al. 2016) was utilized, which relies on an unsupervised clustering method known as superparamagnetic clustering (SPC). The “plot_kselect” function determines the optimized value for the maximum number of nearest neighbours (K-NN) that an individual can possess, with values ranging from 1 to 12, thereby exploring the structure from the fine to the larger scale. The population networks were then visualized using different node colors for each breed.

Subsequently, an analysis was performed using Treemix software (Pickrell and Pritchard 2012). To define the tree’s topology, bootstrap replicates were generated using 500 contiguous SNP blocks (-k 500), exploring 0–10 possible migratory events and five replicates. Migration edges are usually added when 99% of the variation in the data is explained. We used two methods, linear and Evanno, to estimate the best number of migratory events. We did this using a function ‘OptM’ in R software (R Core team 2025), following the approach proposed by Fitak (2021).

Finally, an analysis using ADMIXTURE (Alexander et al. 2009) was carried out to infer the genetic structure and ancestral composition of the populations. For this purpose, the all-breed LD-pruned database was used, and 2–12 possible ancestral populations were considered using 2,000 bootstrap replicates (-B2000). The termination criterion is to stop when the log-likelihood increases by less than 10⁻⁴ between iterations. The optimal k was selected using cross-validation error analysis with the R BITEV2 package (Milanesi et al. 2017).

## Results

### Linkage disequilibrium (LD), diversity and effective population size (Ne)

Before quality control, the average minor allele frequency (MAF) for HV-CN was 0.25 and for Lucerna, it was 0.29. After the quality control editing, we obtained a dataset of 10 HV-CN individuals (4 males and 6 females) containing 77,720 SNP markers. The genotyping rate was 99.82%. Lucerna had 92 individuals (19 males and 73 females) with 77,210 SNP markers and a 99.44% genotyping rate. The average MAF after quality control was 0.28 for HV-CN and 0.31 for Lucerna (see Table 1).

**Table 1:** Diversity estimates in Harton del Valle conservation nucleus (CN) and in the Lucerna breed

Population	SNP Number	MAF	LD (*r*^2^) to 1 Mb	A. dist (kb) to LD	A. dist (kb) LD > 0.3	Ne	HO	HE
Harton del Valle - CN	77,720	0.28	0.28	159.34	145.59	13	0.40	0.34
Lucerna	77,210	0.31	0.17	160.16	109.15	58	0.43	0.41

^Minor allele frequency (MAF), linkage disequilibrium (LD), Average distance (A. dist), effective population size (Ne), observed and expected heterozygosity (HO and HE)^

**Fig. 1:**
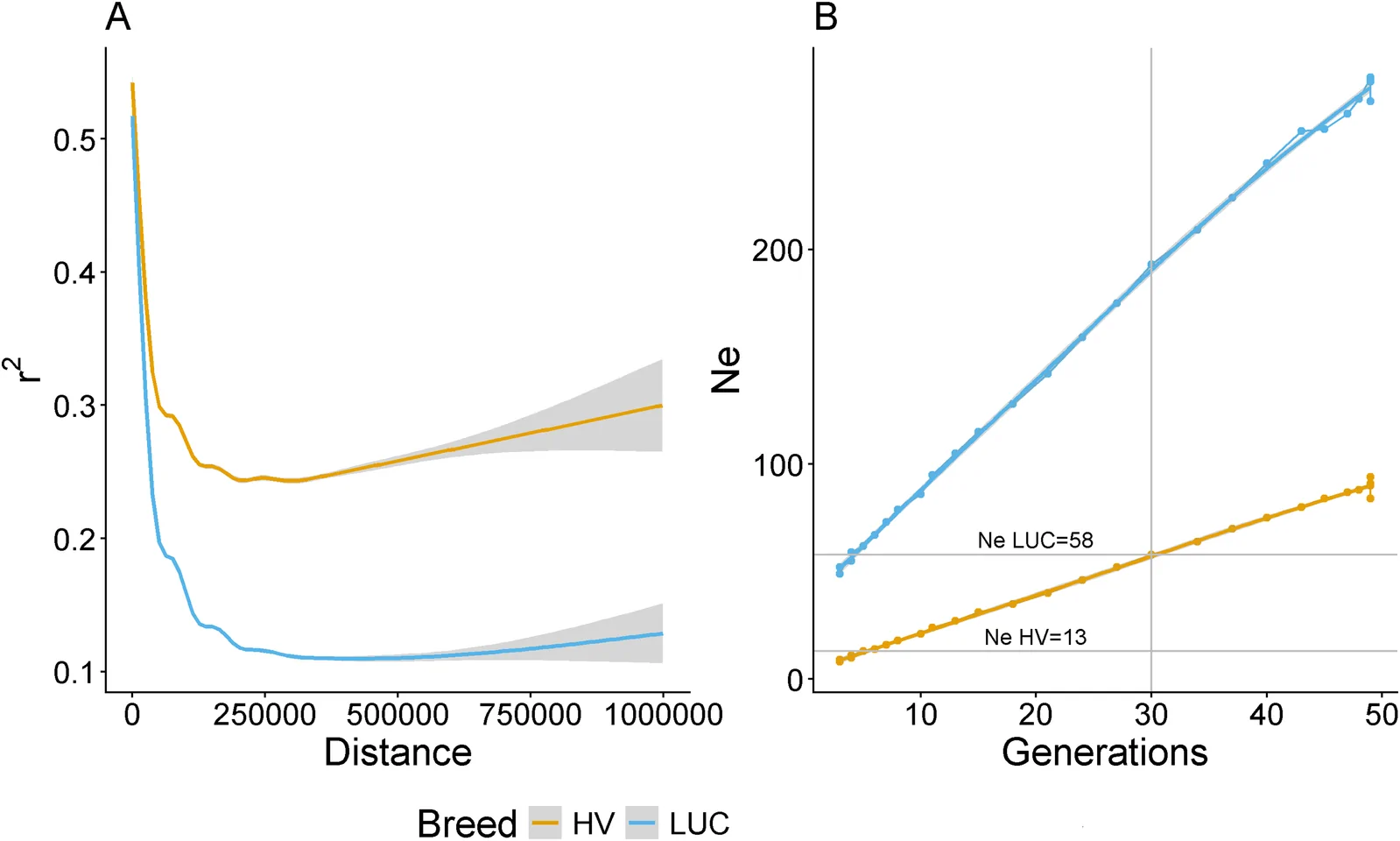
**A** Decay of linkage disequilibrium (r2) by distance (in base pairs) for Harton del Valle (HV) conservation nucleus and Lucerna (LUC); **B** Effective Population Size (Ne) for HV-CN and LUC in the last 50 generations, based on LD

Considering distances less than 1,000 kb, the average linkage disequilibrium (LD) was 0.28, with a mean distance of approximately 159.34 kb for HV-CN and an LD of 0.17 at a mean distance of 160.16 kb for Lucerna (Table 1). Additionally, the average distance at which r² > 0.3 was achieved was 145.59 kb for HV-CN and 109.15 kb for Lucerna. This shows greater persistence of LD (r²) for the HV-CN breed than for Lucerna (Fig. 1A).

The effective population size (Ne) showed significant differences between the two breeds, with Ne of 13 for the HV-CN and Ne of 58 for the Lucerna breed (Table 1), Which makes sense when you consider that only the HV conservation nucleus was included. However, a steady decline has been observed for both populations over the last 50 generations, with the Lucerna breed experiencing a more marked decline (Fig. 1B).

### Runs of homozygosity (ROH) and inbreeding

The analysis of runs of homozygosity (ROH) identified 242 ROH fragments for HV-CN individuals covering approximately 209,900 ± 48,280 kb of the genome on average, meaning that around 8.43 ± 1.94% of each individual’s genome was autozygous. For Lucerna, 2,670 ROH fragments were identified, covering an average of 174,150 ± 9,200 kb per individual, with an average autozygosity of 7.00 ± 0.37% (inbreeding by ROH), as shown in Table 2. The results show that in the HV-CN genome, the highest proportion of homozygosity regions were observed in short segments (ROH1,000–4,000 kb), representing 38.43% of the segments, while the lowest proportion was found in segments of 8,000–12,000 kb, with 15.29%. However, the highest homozygous genome coverage (5.08% of the genome) was for long segments (ROH > 12,000 kb). Similarly, for the Lucerna breed, the largest proportion of segments were short (ROH1,000–4,000 kb), representing 52.02%, and the smallest proportion were segments of 8,000–12,000 kb (10.04%). However, the highest coverage of the homozygous genome (3.00% of the genome) was due to long fragments (> 12,000 kb), indicating recent homozygosity.

**Table 2:** ROH inbreeding Coefficient and ROH distribution in different length segments (1,000–4,000 kb, 4,000–8,000 kb, 8,000–12,000 kb, and > 12,000 kb) for the Lucerna (LUC) and Harton del Valle conservation nucleus (HV-CN)

Breed	Variable	ROH 1,000–4,000 kb	ROH 4,000–8,000 kb	ROH 8,000–12,000 kb	ROH > 12,000 kb	TOTAL ROH
HV-CN	N ROH	93	66	37	46	242
	Proportion of ROH segments (%)	38.43	27.27	15.29	19.01	100
	F_ROH_ – ROH coverage (%)	0.93 ± 0.14	1.45 ± 0.36	1.48 ± 0.24	5.08 ± 1.67	8.43 ± 1.94
LUC	N ROH	1389	710	268	303	2670
	Proportion of ROH segments (%)	52.02	26.59	10.04	11.35	100
	F_ROH_ - ROH coverage (%)	1.5 ± 0.04	1.79 ± 0.08	1.27 ± 0.08	3.00 ± 0.27	7.00 ± 0.37

^Standard deviation (±), Number of ROH segments (N ROH), ROH Inbreeding coefficient (FROH)^

The FIS results for the two populations indicate a tendency toward exogamy. In the case of the HV-CN, a value of -0.17 was obtained, and in Lucerna, a value of -0.05. These results indicate an excess of heterozygotes in both breeds. On the other hand, for HV-CN, Ho was 0.40 and He was 0.34. For Lucerna, Ho was 0.43 and He was 0.41, as shown in Table 1. These results indicate that there is a heterozygous excess in both populations, which is consistent with the tendency toward exogamy obtained in the FIS statistic.

### Genetic structuring, admixture, and treemix analysis

**Fig. 2:**
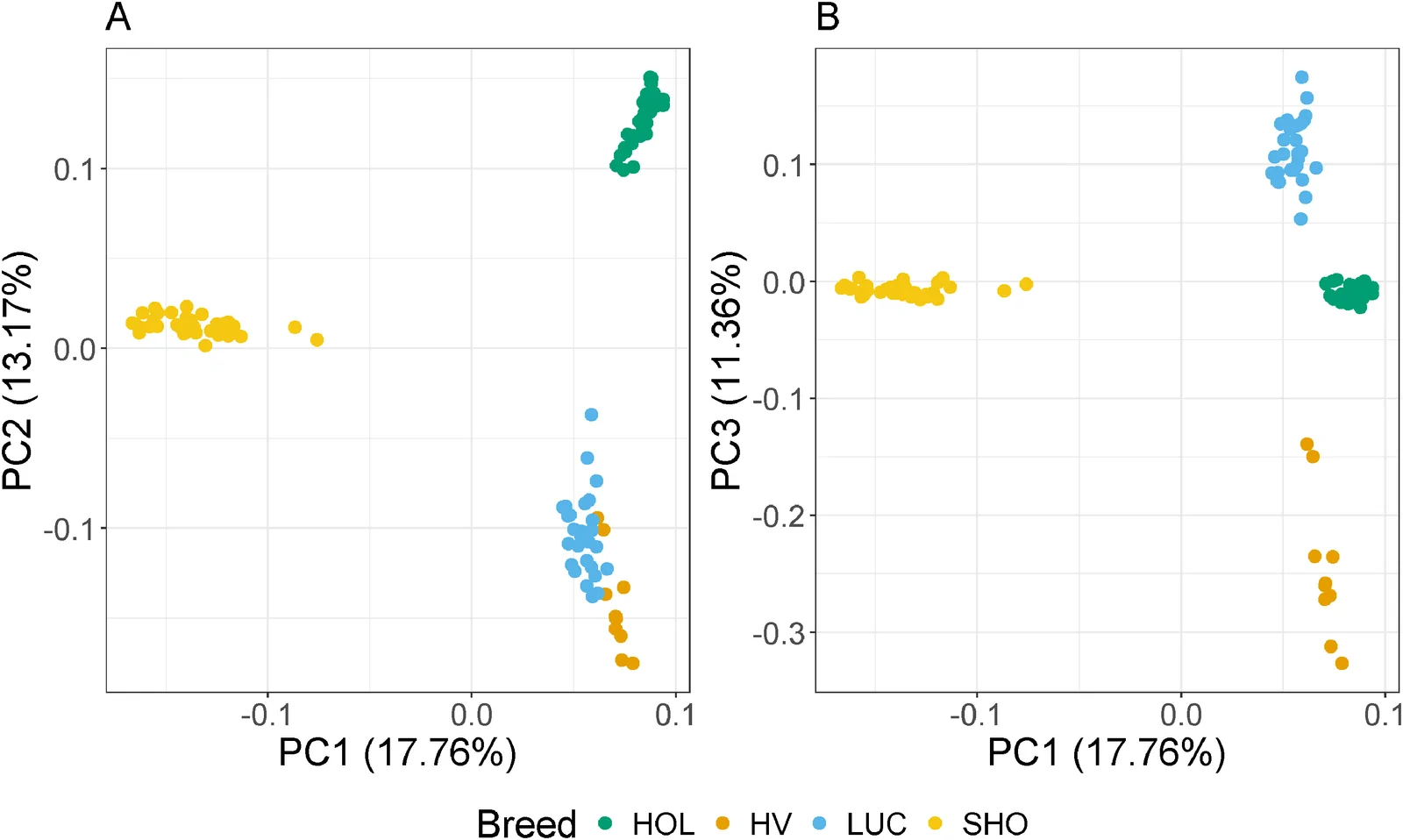
Principal component analysis. (**A**) PC1 vs. PC2; (**B**) PC1 vs. PC3 This is based on the SNP genotypes of the breeds that contributed to the formation of Lucerna. Holstein (HOL), Harton del Valle (HV), Lucerna (LUC), Shorthorn (SHO)

The PCA analysis included the breeds that constitute Lucerna: HV, Holstein, and Shorthorn. The first component explained 17.76% of the total variability, and the second explained 13.17%. Plotting these two components (Fig. 2A) reveal a clear separation between the Holstein and Shorthorn breeds. Lucerna and HV are shown together, indicating greater similarity between these two Colombian Creole populations. This is related to the influence of the HV breed in forming Lucerna and the subsequent selection that stabilized the breed under tropical conditions. When considering the third component, which explained 11.36% of the total variability (Fig. 2B), a greater difference between HV and Lucerna is evident, indicating that, although they share some genetic similarity, there are additional differences that are captured in the third component. Even when performing PCA analysis on databases including only HV and Lucerna, the first component explained 14.16% of the total variability, while the second explained 9.73%. When graphically representing the two components (Supplementary Figure 1), a separation between the two breeds evaluated can be observed, evidencing genetic structuring.

In order to carry out the comparison with other breeds, a database was created that incorporated genetic information from the Brahman (25), Gir (24), Holstein (30), Negra Andaluza (32), Romosinuano (8), and Shorthorn (34) breeds, comprising 110,174 different SNPs and presenting a genotyping rate of 50.48%. Ten HV animals and 30 Lucerna animals were randomly selected from the total database in order to avoid group size bias.

After combining data, applying quality control, and LD-pruning, a database containing 12,404 SNPs and 190 animals were obtained with a genotyping rate of 99.59%. The average MAF values for each breed were calculated using the common genetic markers in the database. The Brahman and Gir breeds had the lowest MAF values, at 0.25 and 0.23, respectively. In contrast, the Holstein breed had an MAF of 0.34, the HV-CN breed had an MAF of 0.32, the Shorthorn breed had an MAF of 0.30, and the Lucerna, Negra Andaluza, and Romosinuano breeds had MAFs of 0.33.

**Fig. 3:**
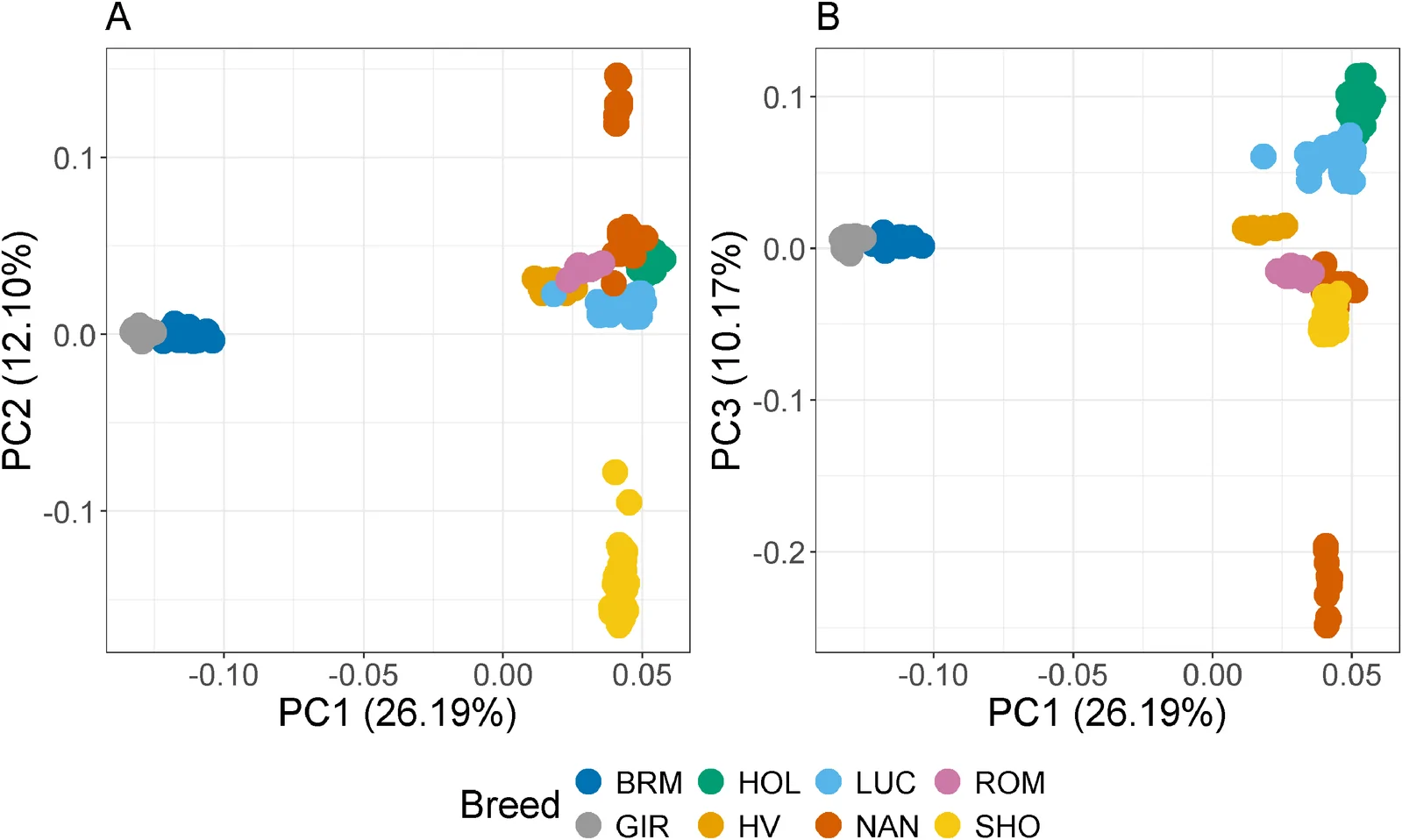
Principal component analysis (**A**) PC1 vs. PC2; (**B**) PC1 vs. PC3 based on SNP genotypes of the Brahman (BRM), Gir (GIR), Holstein (HOL), Harton del Valle (HV), Lucerna (LUC), Negra Andaluza (NAN), Romosinuano (ROM), and Shorthorn (SHO) breeds

In the PCA analysis, which included other breeds, an interesting pattern of separation between the breeds was observed. The first principal component, which explained 26.19% of the variability, showed a clear separation between indicus cattle and other taurine breeds (Fig. 3A). The second component, which explained 12.10% of the total variability, allowed us to observe the Shorthorn group separating at the bottom. In the graph, the Lucerna breed is close to the Colombian HV and Romosinuano breeds.

To clarify the genetic structure, the third component was used, explaining 10.17% of the total variability (Fig. 3B). In this analysis, Lucerna was observed near Holstein and far from HV. Figure 3 generally shows that Lucerna exhibits a distinct grouping from the other breeds. This suggests that Lucerna acts as an independent breed from those that formed it. Lucerna is close to the other Creole and Iberian breeds, as well as Holstein. Additionally, Lucerna is observed to be farther from Shorthorn, indicating a lower contribution to the breed conformation.

The overall FST value for all breeds was 0.15. Slatkin’s paired FST statistics revealed that Lucerna had the highest structuring value with the Gir breed, with an FST of 0.31 and an Nm of 1.62 per generation. Conversely, the lowest paired FST value for Lucerna was with the Holstein breed (FST = 0.10 and Nm = 4.81 per generation). Table 3 shows that the value between the HV and Lucerna breeds was 0.15, reflecting moderate isolation between the two breeds. A value of 0.14 was obtained between Lucerna and Shorthorn. The complete results of Slatkin’s paired FSTs (below the diagonal) and Nm (above the diagonal) are shown in Table 3.

**Table 3:** Slatkin’s paired FST values (below the diagonal) and number of migrants (above the diagonal) between the different cattle breeds evaluated

Breed	BRM	GIR	HOL	HV	LUC	NAN	ROM	SHO
Brahman (BRM)		9.41	1.92	1.65	1.95	2.01	1.67	1.74
Gir (GIR)	0.05		1.60	1.33	1.62	1.67	1.32	1.46
Holstein (HOL)	0.26	0.31		2.89	4.81	4.51	3.49	3.27
Hartón del Valle (HV)	0.30	0.37	0.17		3.29	3.15	2.67	2.30
Lucerna (LUC)	0.26	0.31	0.10	0.15		4.38	3.51	3.47
Negra Andaluza (NAN)	0.25	0.30	0.11	0.16	0.11		4.64	3.27
Romosinuano (ROM)	0.30	0.38	0.14	0.19	0.14	0.11		2.63
Shorthorn (SHO)	0.29	0.34	0.15	0.22	0.14	0.15	0.19	

**Fig. 4:**
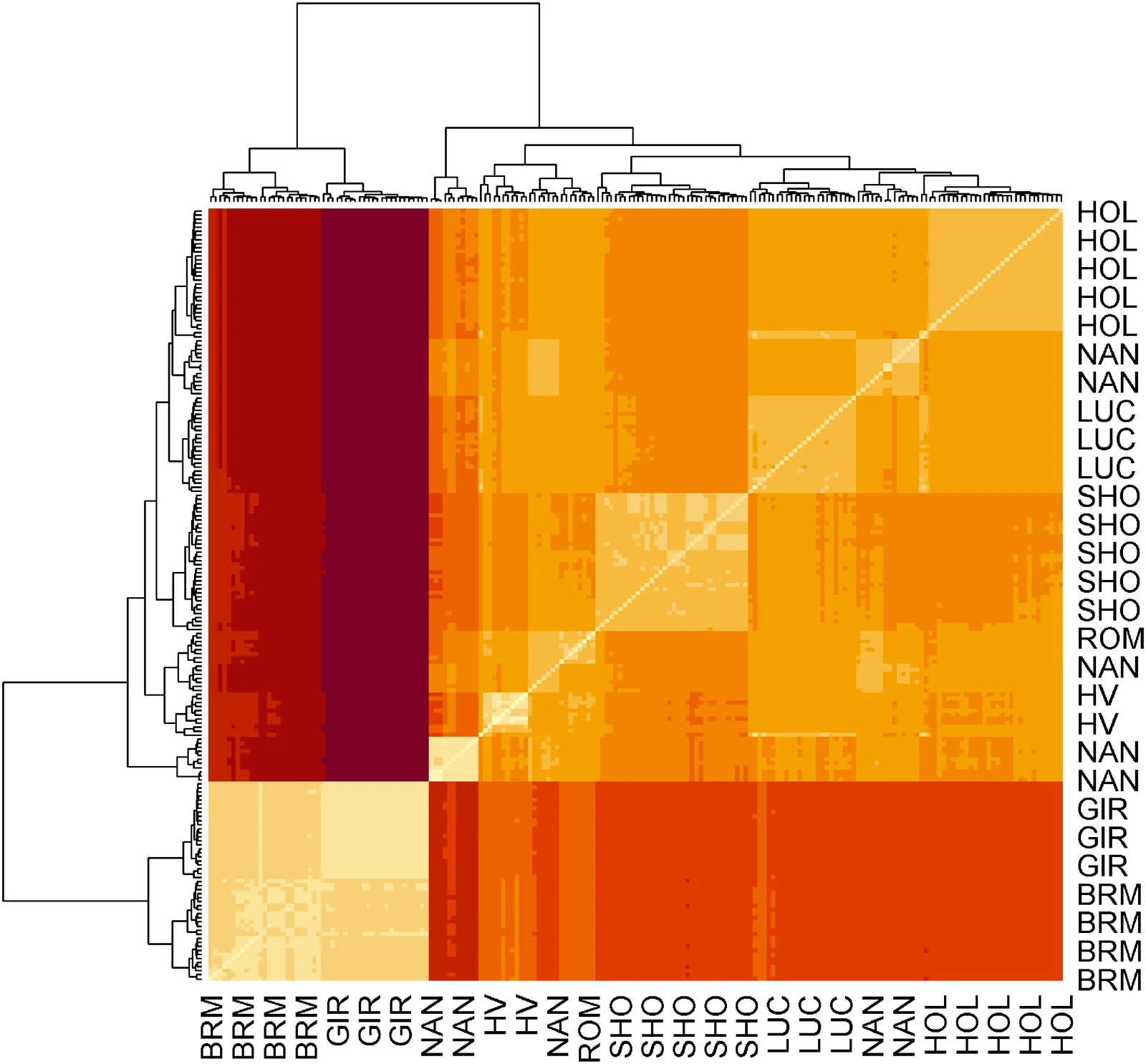
Dendrogram and heat map based on IBS distances of the evaluated breeds. The breeds evaluated are Brahman (BRM), GIR, Negra Andaluza (NAN), Harton del Valle (HV), Romosinuano (ROM), Shorthorn (SHO), Lucerna (LUC) and Holstein (HOL)

When analyzing IBS distances using a dendrogram with a heat map (Fig. 4), two groups can be seen: one composed of Indicus cattle, including Brahman and Gir, which are separated into subgroups, and another composed of taurus breeds. Lucerna forms a clear, homogeneous group, as do Holstein, HV and Shorthorn. It can be concluded that Lucerna behaves as a distinct breed. The graph also shows that the Negra Andaluza (NAN) breed forms two groups, as was evident in the PCA analysis.

**Fig. 5:**
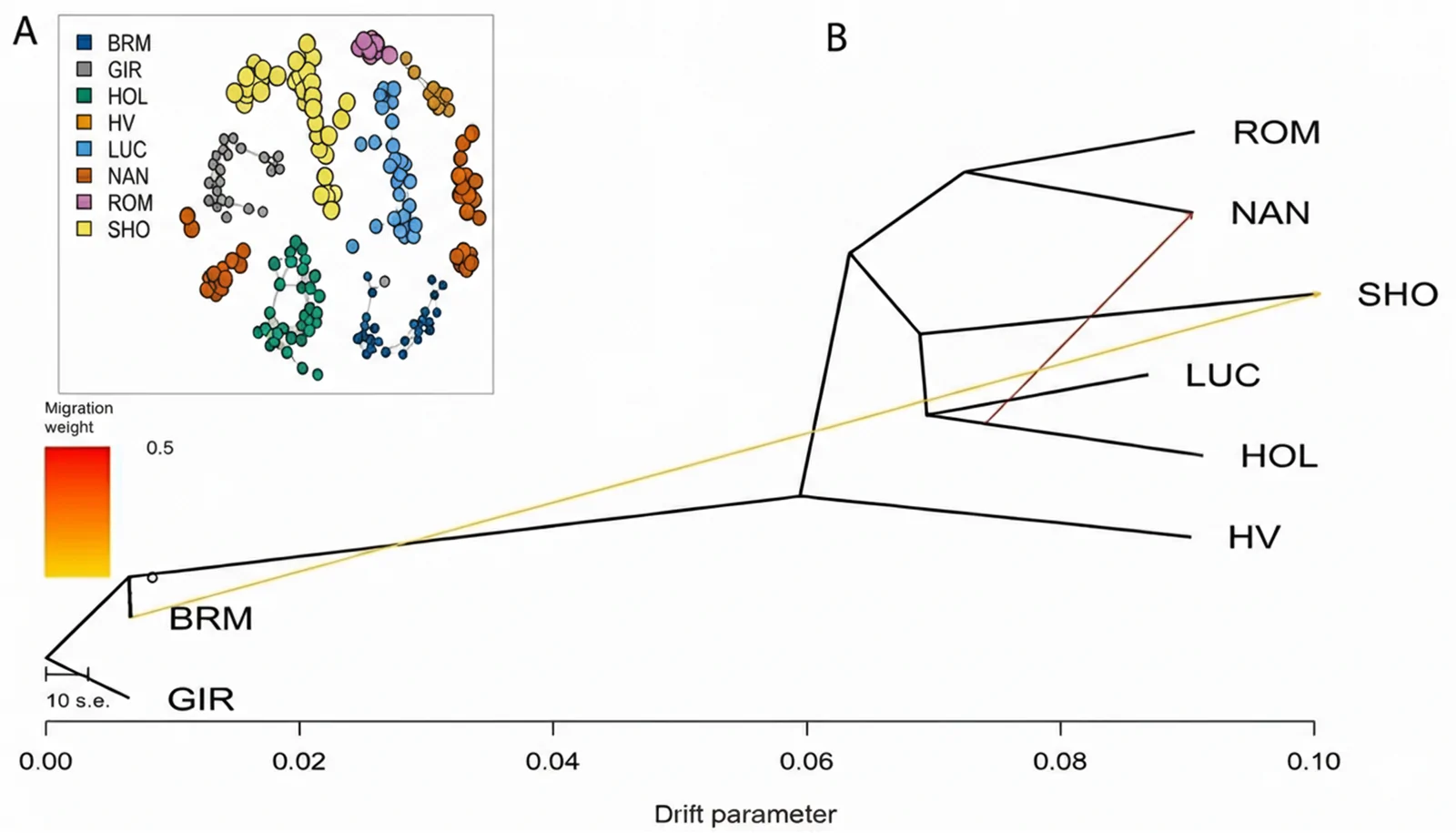
**A**) Netview population structure based on Nei genetic distances between the different bovine breeds. The best number of nearest neighbors (K-NN) = 7; **B**) Treemix representation of bovine populations evaluated

Visualizing the population structure based on Nei’s genetic distances allowed us to generate mutual nearest neighbor graphs (Fig. 5A). These graphs show the connections and gene flow between Lucerna and other breeds according to the best estimate of k, which was approximately k7 (see Supplementary Figure 2A). The graph shows the separation of the Lucerna population, indicating a lack of genetic flow to other breeds. In contrast, the Treemix analysis using the linear method allowed us to determine the optimal number of migratory events between breeds. This analysis suggests that there were two significant migration episodes in the recent history of these populations (see Supplementary Figure 2B). However, it is important to consider that due to the small sample size of some breeds, Treemix inference should be considered with caution. Figure 5B shows the relationship between populations and migratory events. Once again, Lucerna is observed to be close to Holstein without significant migration events from the breeds that composed Lucerna.

**Fig. 6:**
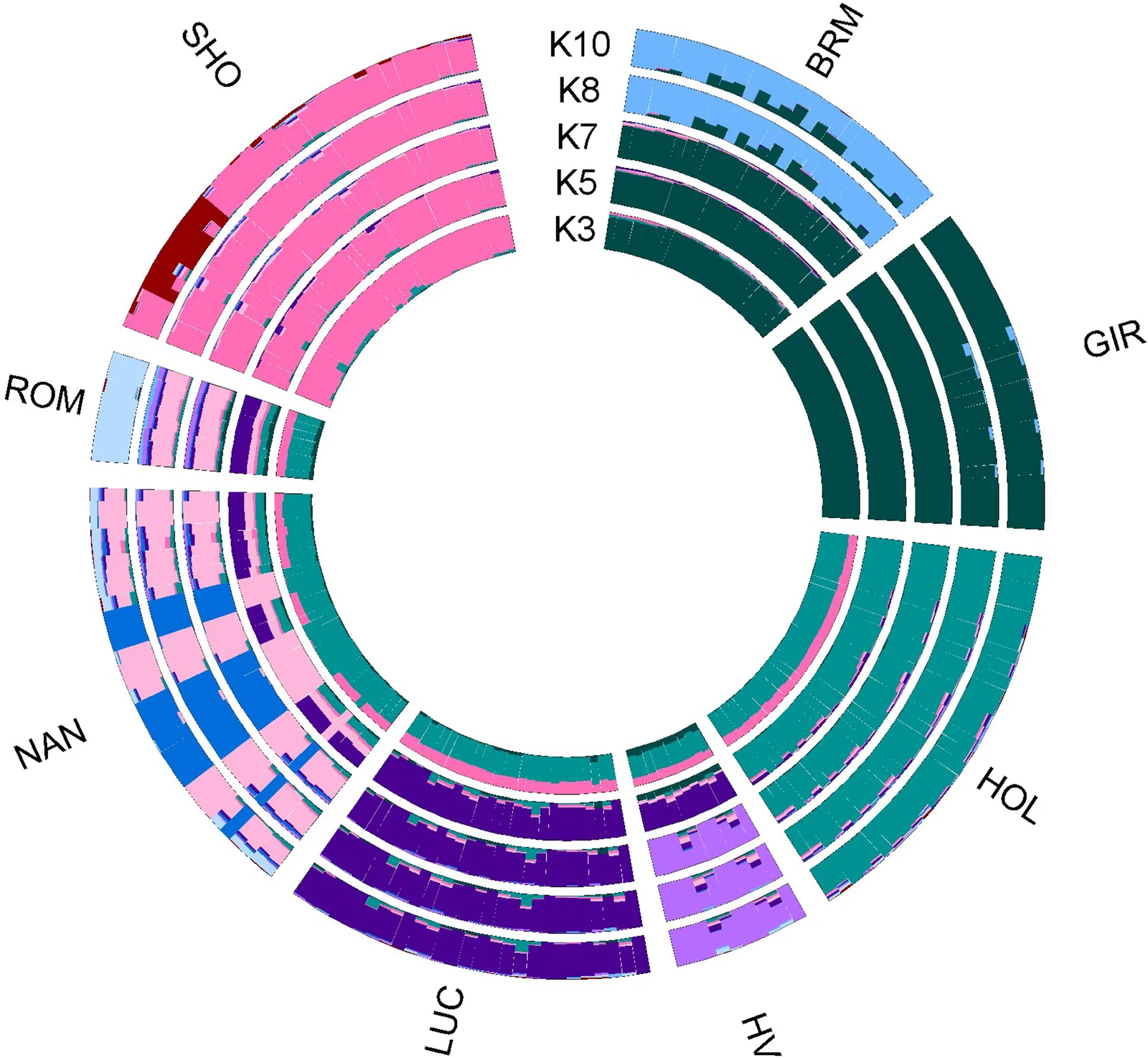
Circular admixture plot of bovine breeds, including Colombian creole cattle for K = 3, 5, 7, 8 and 10 hypothetical ancestral populations (best K = 7). Each bar indicated an individual and each color the membership to a particular cluster: Brahman (BRM), GIR, Holstein (HOL), Harton del Valle (HV), Lucerna (LUC), Negra Andaluza (NAN), Romosinuano (ROM) y Shorthorn (SHO)

Finally, the Admixture analysis (Fig. 6) shows the results for all evaluated breeds, identifying k = 7 as the best based on cross-validation error (see Supplementary Figure 2C). Figure 6 shows the results for k-values between 3 and 12. With k = 3, a cluster representing Indicus cattle can be observed, along with two different clusters for taurine cattle. When evaluating K = 5, the Brahman and Gir breeds are still in a single cluster. Additionally, a cluster is identified for the Holstein breed. The HV-CN and Lucerna breeds have a similar composition and are observed in one cluster. NAN showed two distinct groups, one of which was similar to Romosinuano. The Shorthorn breed was grouped in a separate cluster but also showed some similarity to the other taurine breed groups. When plotting K = 7, the Brahman and Gir breeds are seen to form a single group, while the Lucerna breed forms a different cluster. Using K = 8, Brahman and Gir are separated into individual clusters. Independent clusters were also identified for Holstein, Shorthorn, HV-CN, Lucerna, and Romosinuano. NAN continued to show two distinct groups, though with fewer similarities to Romosinuano this instance. Finally, with K = 10, separate clusters were observed. In this case, Romosinuano was in a single cluster, and Shorthorn was in two different clusters.

## Discussion

Creole cattle are notable for their unique genetic diversity, which has been shaped by a combination of migration, genetic drift, and natural and artificial selection over successive generations. This has enabled them to adapt to specific environmental conditions, rendering them a valuable genetic resource for future production in tropical regions. Given their importance to agriculture and the various benefits they provide, it is essential that their genetic diversity is studied and understood (Polak et al. 2022). In the case of Lucerne cattle, this information is necessary to guide breeding and selection programs that enable breeders to sustainably utilize this valuable genetic resource.

After quality control, the MAF for the HV-CN and Lucerna breeds was 0.28 and 0.31, respectively. This suggests greater genetic diversity in Lucerna or a greater number of segregating markers, which seems reasonable given that only a conservation population was used for HV, rather than a representative sample of the population. These MAF values are similar to those of other Creole breeds, such as Blanco Orejinegro (BON) and Romosinuano cattle, which have an average MAF of 0.27 (Bejarano et al. 2018). Other studies have reported lower values for the Chino Santandereano and Romosinuano breeds, with MAF values of 0.34 and 0.38, respectively (Ocampo-Gallego et al. 2021). A recent study on the BON breed reported an average MAF of 0.32 (Caivio-Nasner et al. 2021). In indicine breeds, an MAF value of 0.24 is observed in the Gir breed (Garcia et al. 2023). This is understandable due to the ascertainment bias of the chip used in this study. The chip was designed for taurine cattle; therefore, many non-segregating markers are expected in indicine breeds.

LD analysis revealed differences between the HV-CN and Lucerna breeds. The average LD up to a distance of 1,000 kb was significantly higher in the HV conservation nucleus (0.28) than in the Lucerna breed (0.17). These findings suggest greater linkage persistence in HV-CN than in Lucerna, meaning there is probably more homozygosity and less diversity in HV-CN, which seems reasonable given that only a conservation population was used. The Fig. 1A showing linkage disequilibrium decay indicates that Lucerna decays faster than HV-CN, suggesting greater diversity. The r² values in this study are higher than those reported by Ocampo-Gallego et al. (2021), for the Chino Santandereano Creole breed, where r² was equal to 0.13 at the same distance but with lower genotyping density (26 K). LD was also reported in the BON breed considering a distance of up to 1,000 kb, where LD was 0.21, but with a lower genotyping density of 50 K (Caivio-Nasner et al. 2021). A study conducted by Bejarano et al. (2018), shows similar results to this study at a distance of 1,000 kb, r² was 0.23 for the Romosinuano breed and 0.19 for the BON breed.

On average, an r² > 0.3 was obtained at a distance of 145.59 kb for HV-CN and 109.15 kb for Lucerna. Assuming a genome size of 2489,385 kb, it is suggested that the persistence of linkage to QTLs for genomic evaluation would require a genotyping density of at least 17,097 SNP markers for HV-CN and 22,805 for Lucerna. These results show a faster LD decay and are lower than those reported in Chino Santandereano, where an LD greater than 0.3 was found at an average distance of 340.3 kb (Ocampo Gallego et al. 2021). Lower values were found in BON cattle, where r² > 0.3 was observed at a distance of 77.43 kb (Caivio-Nasner et al. 2021). Bejarano et al. (2018) reported distances of 70 kb for BON and 100 kb for Romosinuano for the same statistic. Although the distances at which an r² value greater than 0.3 is reached provide information on the persistence of genetic linkage, it is important to note that these values may vary depending on the population’s size and structure, genotyping density, quality control parameters and genetic diversity.

A downward trend in effective population size (Ne) was observed across generations in both breeds. The Lucerna breed had a current Ne value of 58, indicating a relatively large effective population size. In contrast, the HV-CN had an Ne value of 13, with a significant decline in effective population size across generations, which is significant given that it was a single conservation herd. This decrease in Ne over time may result from several factors, such as a reduction in the number of breeding animals or bottleneck events that limit genetic variability in the population. Compared to Creole cattle, a higher Ne value of 123 was reported in the BON breed (Caivio-Nasner et al. 2021). It is important to note that the genotyping density was different and this breed has the largest Creole cattle inventory in the country. This contributes to a larger Ne due to the greater number of breeders and wider genetic variability. In the Chino Santandereano breed, Ne was reported as 32 (Ocampo-Gallego et al. 2021). A study on Gir cattle reported a Ne value of 11 in Zebu breeds (Garcia et al. 2023).

On the other hand, a study of a Spanish Holstein population determined Ne using genealogical information and complete genome data. The results were consistent and ranged from 66 to 79 (Rodríguez-Ramilo et al. 2015). According to the FAO recommendation (FAO 2013), the optimal number of animals per generation is around 50 to achieve the desired effective population size. However, a larger number of animals is preferable to ensure the long-term survival of the population. The Lucerna breed, with an Ne of 59, exceeds the desired size, suggesting that its genetic diversity and selection capacity are sufficient for its conservation and use. Conversely, the HV breed conservation population has an effective population size of only 13 individuals, which is smaller than the desired size for a conservation nucleus. It is important to note, however, that this value represents only the conservation nucleus and not the HV breed as a whole. Therefore, future research should include a larger number of herds to better understand the breed’s genetic diversity and provide additional recommendations for the conservation program.

This study identified 242 regions of homozygosity (ROH) fragments in HV-CN, representing an average of 24.2 ROH segments per individual. The total length of these fragments was 209,900 kb on average, equivalent to 8.43% autozygosity or 7,940 kb per individual. For the Lucerna breed, 2,670 ROH fragments were identified, representing an average of 29.02 regions per individual. The average sum of all lengths covered 174,150 kb, indicating an average length of 5,710 kb per individual and equivalent to 7.00% inbreeding. The HV-CN has a higher level of inbreeding than the Lucerna breed, consistent with previously reported lower diversity. Similar values were reported in the BON Creole breed, where ROH regions covered an average of 170,360 kb per individual with an average homozygosity of 6.78% (Caivio-Nasner et al. 2021). These values are lower than those of the HV-CN in this study. Ocampo Gallego et al. (2021) reported a lower result than both breeds evaluated in this study, where ROH regions covered 134,560 kb per individual, and 5.36% of the genome was autozygous. Bjelland et al. (2013) reported ROH inbreeding of 3.8% in Holstein. Both breeds showed significant autozygosity; therefore, inbreeding control programs should be maintained to prevent excessive consanguinity and allow for the introduction of new genes through crossbreeding with other populations.

Both HV-CN and Lucerna cattle showed a greater contribution to inbreeding with long ROH (> 12,000 kb), indicating recent inbreeding (within the last 4.16 generations). This may be related to processes such as population reduction, recent selection and the absence of inbreeding control programs. Previous studies on Chino Santandereano and Blanco Orejinegro cattle reported similar results, showing a higher proportion of recent autozygosity in Creole cattle in Colombia (Caivio-Nasner et al. 2021; Ocampo-Gallego et al. 2021).

The observed (Ho) and expected heterozygosity (He) of the HV-CN (Ho = 0.40, He = 0.34) and Lucerna (Ho = 0.43, He = 0.41) breeds were analyzed. The results show that the Lucerna breed has greater genetic variability and a higher proportion of heterozygous alleles than the HV-CN. However, the results also reveal that both breeds exhibit greater observed heterozygosity than expected heterozygosity, indicating an excess of heterozygotes in both populations. The FIS statistics were negative for both breeds (-0.017 for HV-CN and − 0.05 for LUC), indicating a tendency toward exogamy. In the case of HV-CN, this coincides with the introduction of breeding stock from other farms and the consequent contribution of new alleles to the population. For Lucerna, this may be related to the good reproductive and record management practices that farmers have implemented since developing the breed.

A study evaluating eight commercial subpopulations of Romosinuano Creole cattle revealed that two subpopulations had similar results. These populations had excess heterozygotes and negative FIS values (-0.006 and − 0.088, respectively) (Bejarano et al. 2012). However, a study involving several Creole breeds, including Lucerna and HV, found the opposite. The average Ho value for Creole breeds was higher, at 0.71 and the average He value was higher, at 0.80 (Martínez et al. 2012). This study used microsatellite markers, which tend to be more polymorphic than SNPs and have a higher mutation rate.

The PCA analysis of the Lucerna breeds separated the Shorthorn breed from the others in the first component. This may be related to the Shorthorn’s origin and evolutionary history. Although it was used for breed formation, it has subsequently seen little use in Colombian livestock production. The second component separated the Holstein breed from the Creole breeds (Lucerna and HV). It is important to mention that the Creole breeds, HV and Lucerna have a similar evolutionary history and their ancestry is partly derived from Iberian cattle. This differentiates them from the other evaluated breeds (Martínez et al. 2012).

As expected, given their marked differences in geographical origin, evolutionary history, and phenotypic characteristics (Verdugo et al. 2019; Hu et al. 2020), the first component in the analysis including all breeds clearly separated taurine cattle from indicus cattle. The second component shows the separation of the Shorthorn group from the other taurine breeds, attributed to their evolutionary history. Interestingly, Lucerna is close to Holstein and the Creole breeds HV and Romosinuano in this component. This shows its proximity to the Negra Andaluza group, which belongs to Iberian cattle. This is consistent with the origin of Creole breeds from Iberian cattle during the conquest (Martínez et al. 2012). The third component shows the separation of each breed, placing Lucerna close to Holstein. However, admixture analysis revealed that only 6.33% of Lucerna corresponds to the Holstein cluster.

Analyzing all breeds using Slatkin’s paired FSTs supports and complements the findings from previous PCA analyses. Taurine and Indicus cattle breeds are distinctly separated due to lower gene flow causing population structuring. Netview analysis also supports this. In the PCA analysis involving the breeds that comprise Lucerna, Holstein was found to be close to Lucerna, which was close to HV. While some previous studies using different markers and methods have reported a clear separation between certain American Creole breeds and specialized breeds, such as Holsteins (Martínez et al. 2012; Giovambattista et al. 2013). It is important to mention that this study used Slatkin-paired FSTs and examined all breeds, including those that comprise Lucerna. Thus, the results of this analysis may provide a complementary perspective by highlighting the distinct genomic profile of Creole breeds compared to specialized breeds.

The Admixture analysis of all breeds allows us to assume a number of hypothetical ancestral populations ranging from 7 to 8, based on the cross-validation error (see supplementary Figure 2C). When we graph the results at K = 3, we see that the indicus breeds form a single cluster, while the taurine breeds form two groups: one for British cattle and one for Iberian cattle. As we continue the analysis, K = 5 groups the Bos indicus breeds (Brahman and Gir) into a single cluster. Meanwhile, Lucerna and HV appear to share the same cluster. At K = 7, the indicine breeds remained in one group, while Lucerna and HV had their own clusters. Lucerna behaved as a distinct cluster breed, with slight mixing with the other breeds, more than with the cluster associated with Holsteins. With K = 8, the separation of the indicus breeds is already apparent. These results suggest for the first time that Lucerna has its own genomic profile and constitutes an independent breed, consistent with the FAO report (Ajmone-Marsan et al. 2023).

The admixture analysis revealed a percentage of introgression from other breeds within the Lucerna cluster. Although the Treemix analysis did not show any migration events for the Lucerna breed, it was possible to estimate that, on average 2.17% of Lucerna animals’ composition corresponded to the indicus cluster, primarily Gir (1.55%). This is understandable since Gir is a widely used dairy cattle breed in Valle del Cauca, so gene introgression may have occurred. Conversely, the Holstein component may represent the most significant introgression, averaging 6.33%. This is primarily because purebred and crossbred animals with specialized breeds, such as Holstein, are used in tropical dairy farming, especially in the sampled herds. Therefore, it is possible that some introgression occurred at some point, in addition to the association with the participation of Holstein in the formation of Lucerna.

In the case of HV-CN, 2.02% of the mixture corresponded to Indicus, primarily Brahman (1.21%). Some Holstein (0.20%) and Lucerna (4.16%) were also present. A study by Martínez et al. (2012) that used microsatellite markers, reported indicine, British, and Iberian cattle contributions to the HV and Lucerna Creole breeds. This finding is consistent with the results of this study. Another study that used Structure to determine the genetic structure of populations observed a small amount of indicine cattle admixture within the HV cluster (Vargas et al. 2016). Conversely, a recent study involving HV cattle revealed evidence of recent introgression within the breed, indicating that some animals are not fully purebred (Martinez et al. 2023).

Finally, although Lucerna is a synthetic breed, its genetic and phenotypic traits have stabilized, placing it in the independent breed category. It has a genomic footprint and minimal introgression from other breeds in the evaluated population. Additionally, Lucerna diversity is comparable to that of other specialized breeds, establishing the breed’s potential for use in genetic improvement programs. Furthermore, the HV-CN requires more comprehensive analysis and the need to incorporate greater diversity into the conservation nucleus. To achieve this aim, it is necessary to assess the genomic diversity present in the country’s commercial herds. In addition, it is essential to continuously monitor genetic diversity to quickly detect potential problems and provide practical recommendations for the conservation of the breed.

## Supplementary Information

Below is the link to the electronic supplementary material.

Supplementary Material 1

## Data Availability

The data sets generated or analyzed during the current study, or both, are available from the corresponding author upon reasonable request.
