# Supplementary material for: Genomic relationships, diversity and admixture in the Lucerna breed of Colombia
Source: Trop Anim Health Prod. 2026 Aug 10;58(7):481. doi: 10.1007/s11250-026-05227-y (PMC13457441; doi:10.1007/s11250-026-05227-y)
Supplement: Supplementary file 1 [file 11250_2026_5227_MOESM1_ESM.docx]

**Supplementary information**

**Supplementary figure 1:** Principal component analysis (PC1 vs. PC2) based on SNP genotypes in the Hartón del Valle (HV) and Lucerna (LUC) breeds.

**Supplementary figure 2:** A) Plot of the number of detected clusters across k for Netviewr; B) plot of optimal migration edges based on a piecewise linear (change point), bent cable (alpha), simple exponential or non-linear least squares (threshold=5%) models; C) Cross-validation error for k from 2 to 12 hypothetical populations for admixture analysis.
